# Pattern-Dependent Response Modulations in Motion-Sensitive Visual Interneurons—A Model Study

**DOI:** 10.1371/journal.pone.0021488

**Published:** 2011-07-08

**Authors:** Hanno Gerd Meyer, Jens Peter Lindemann, Martin Egelhaaf

**Affiliations:** 1 Department of Neurobiology, Bielefeld University, Bielefeld, Germany; 2 CITEC, Bielefeld University, Bielefeld, Germany; Max-Planck Institute of Neurobiology, Germany

## Abstract

Even if a stimulus pattern moves at a constant velocity across the receptive field of motion-sensitive neurons, such as lobula plate tangential cells (LPTCs) of flies, the response amplitude modulates over time. The amplitude of these response modulations is related to local pattern properties of the moving retinal image. On the one hand, pattern-dependent response modulations have previously been interpreted as '*pattern-noise*', because they deteriorate the neuron's ability to provide unambiguous velocity information. On the other hand, these modulations might also provide the system with valuable information about the textural properties of the environment. We analyzed the influence of the size and shape of receptive fields by simulations of four versions of LPTC models consisting of arrays of elementary motion detectors of the correlation type (EMDs). These models have previously been suggested to account for many aspects of LPTC response properties. Pattern-dependent response modulations decrease with an increasing number of EMDs included in the receptive field of the LPTC models, since spatial changes within the visual field are smoothed out by the summation of spatially displaced EMD responses. This effect depends on the shape of the receptive field, being the more pronounced - for a given total size - the more elongated the receptive field is along the direction of motion. Large elongated receptive fields improve the quality of velocity signals. However, if motion signals need to be localized the velocity coding is only poor but the signal provides – potentially useful – local pattern information. These modelling results suggest that motion vision by correlation type movement detectors is subject to uncertainty: you cannot obtain both an unambiguous and a localized velocity signal from the output of a single cell. Hence, the size and shape of receptive fields of motion sensitive neurons should be matched to their potential computational task.

## Introduction

**Figure 1:**
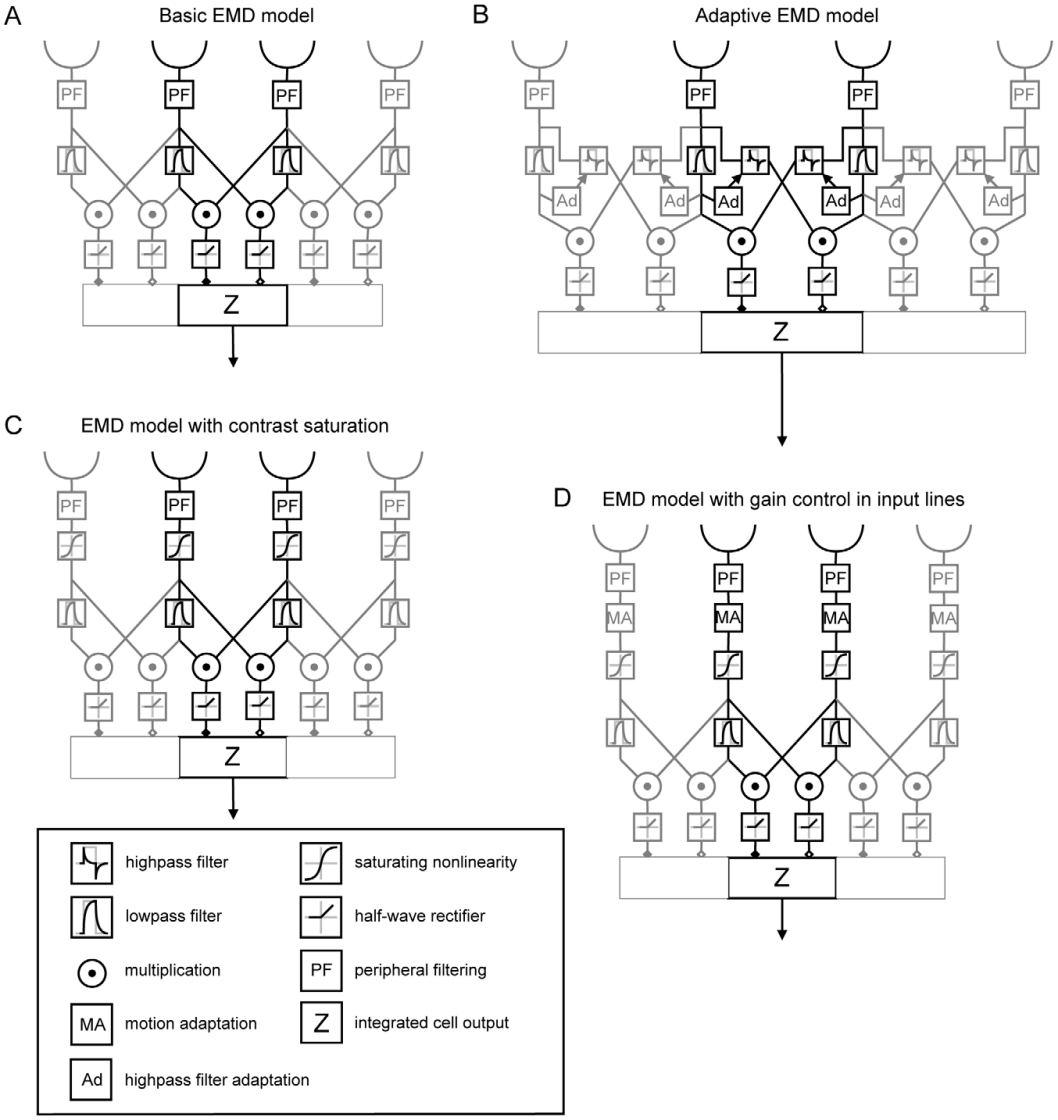
EMD array models used for simulation of LPTC response. (A) **Basic EMD model** including peripheral filtering (*PF*) in the input stage (see Methods and Materials). Signals from each receptor are delayed via the phase delay of a temporal first-order low-pass filter, multiplied and half-wave rectified. Integration of signals in the output cell *Z* is performed according to the *gain control* model [13]. (B) **Adaptive EMD model** extended with a first-order high-pass filter in the cross-arms of the half-detectors. The time-constant of the high-pass filter is adjusted according to the rate of change of the corresponding low-pass signal [14]. (C) **EMD model with contrast saturation** during early visual processing in the input stage. Saturating non-linearities are included to mimic contrast saturation during early visual processing [15]. (D) **EMD model with gain control** **in the input lines** [8]. The input from each receptor channel is divided by the *mean absolute deviation* (see Methods and Materials) in order to control the gain in the input lines.

During locomotion animals continually encounter spatiotemporal changes in their habitat. These changes are reflected in the retinal input and depend in a characteristic way on the animal's self-motion as well as the three-dimensional layout and textural properties of the environment. Hence, to efficiently control locomotion, the nervous system is required to extract behaviorally relevant information from this ever changing retinal input. It has been shown, that the extraction of *visual motion* cues from optic flow (i.e. the field of retinal image velocities) is involved in motor control of a variety of species [1]. In the visual system of flies retinal image motion is processed by about 60 anatomically identified motion-sensitive interneurons, the so called *lobula plate tangential cells* (LPTCs). The processing of motion information by LPTCs is supposed to be relevant in the context of flight stabilization, object detection, visual odometry or spatial navigation [2]. LPTCs respond to visual motion in large parts of the visual field in a direction-selective way, being excited by motion in their *preferred direction* and inhibited by motion in the opposite direction (their so-called *anti-preferred direction*). LPTCs differ in the location and size of their receptive fields. Accordingly they spatially pool the responses of different numbers of retinotopically organized movement sensitive elements from different regions of the visual field. These local motion sensitive elements can be modelled by correlation-type elementary motion detectors (EMDs/Fig. 1). For these EMDs input of at least two spatially separated photoreceptor-channels is required to differentiate directed motion from stationary brightness changes. The delayed signal of one retinal input channel interacts in a multiplicative way with the signal of a neighbouring input channel [3]–[5].

Although natural images share, on average, a typical spatial frequency spectrum [6], the local spatial structure and contrast of individual images may vary strongly. As a consequence, if a natural image moves at a constant velocity across the receptive field of LPTCs, the response amplitude is usually not constant but may modulate over time in a pattern-dependent fashion. Because of these pattern-dependent modulations it is not easily possible to infer the time course of pattern velocity from such neuronal signals. Pattern-dependent modulations have, therefore, been referred to as 'pattern noise', because they deteriorate the neuron's ability to provide unambiguous velocity information [7]. These modulations are also reflected in LPTC models with EMDs as their input channels [8]–[10].

Different functional modifications of the model have been proposed to reduce pattern-dependent modulations and thus to improve the coding of pattern velocity. These modifications include band-pass filtering, compressive/saturating non-linearities in the peripheral visual system as well as motion adaptation and spatial integration of EMDs (Fig.1; [11], [8], [10]). The latter is accomplished by the dendritic integration by LPTCs. The consequences of this integration strongly depend on the size of the receptive fields and their spatial sensitivity distributions. An increasing number of retinotopic inputs included in the receptive field leads to a decrease of pattern-dependent modulations, since spatial changes within the visual field are smoothed out by the summation over many spatially displaced input signals [4], [12]. Whereas dendritic integration improves the accuracy of velocity estimation, the ability of the system to localize movements in the visual field is reduced. The limited size and specific spatial location of the receptive fields of LPTCs indicates that they may provide functionally relevant information about the spatial structure of local features during movements of the animal. However, they do this at the expense of the quality of the velocity signal.

In the current study the influence of the size and shape of receptive fields on the amplitude of pattern-dependent modulations was analyzed systematically by simulations of arrays of four different versions of EMDs which have previously been employed to explain LPTC responses (Fig.1). High dynamic range natural panoramic images moving at constant velocity served as input data. We find that receptive field size and shape influences the pattern-dependent modulations to a great extent. However, large receptive fields deteriorate the ability of the system to localize movements in the visual field, hinting at a trade-off between the quality of velocity signals and their ability to localize moving textures.

## Results

To quantify the influence of changes in the receptive fields on the amplitude of pattern-dependent response modulations LPTC models were analyzed. Models of spatial arrays of four different versions of EMDs were used in computer simulations. These models reflect the fly motion vision pathway from the compound eye to the LPTCs (Fig. 1). Model simulations allow the variations of size and shape of receptive fields, which would be impossible to modify in the '*hard-wired'* visual system of a living animal. The four models used for simulation of LPTC responses have been recently proposed and differ in their internal computational structure emulating different functional aspects of information processing in the visual system of flies (see Methods and Materials). (i) The basic EMD model (Fig.1A) includes temporal filters mimicking dynamic cell properties in the lamina and the retina, correlation-type EMDs and non-linear integration by the model LPTC [13]. (ii) The adaptive EMD model (Fig.1B) includes an additional high-pass filter in the cross-arms of the EMDs. The high-pass filter time-constant is adjusted depending on LPTC activity [14]. (iii) The EMD model with contrast saturation (Fig.1C) elaborates the basic model by including compressive saturating non-linearities in the early visual processing stages [15]. (iv) In the EMD model with gain control in the input lines (Fig.1D) a mechanism for controlling the gain in the input lines of the EMDs is implemented [8].

One class of LPTCs, the HS-cells, respond best to horizontal wide field motion. In the blowfly three HS cells were characterized that respond to horizontal front-to-back motion in the dorsal part (HSN; HS-north), the equatorial part (HSE; HS-equatorial) and the ventral part of the ipsilateral visual field (HSS; HS-south), respectively [16]–[18]. To compare the influence of changes in the receptive fields on the amplitude of pattern-dependent response modulations with a physiologically plausible model, we simulated the responses of the model EMD arrays according to an estimate of an HSE cell receptive field ([19]; see also Methods and Materials for details).

**Figure 2:**
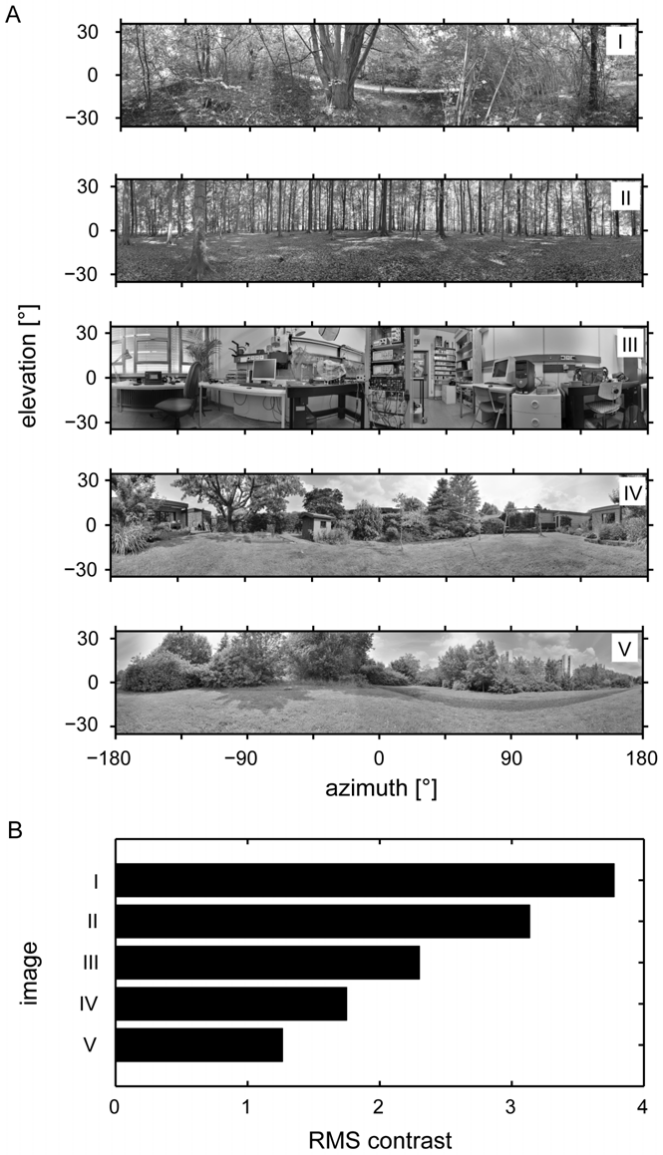
High dynamic range panorama images. (A) Five different panoramic high dynamic range photographs used as input datasets. Images have been normalized, gamma corrected and reduced to 8-bit dynamic range for reproduction. (B) Global root mean square (RMS/see Methods and Materials) contrast for each image. RMS contrast varies considerably between images.

Under natural circumstances flying insects are confronted with contrast and luminance values widely exceeding the ranges that can be represented by photographic images. Consequently five different high dynamic range [20] natural panoramic images, taken in different habitats of flies and varying in contrast and spatial composition, formed the input data sets of the simulations (Fig. 2, see Methods and Materials). Visual motion was simulated by uniform horizontal motion of the panorama images in the preferred direction of the model LPTCs.

### Contrast dependent response modulations of LPTC models

**Figure 3:**
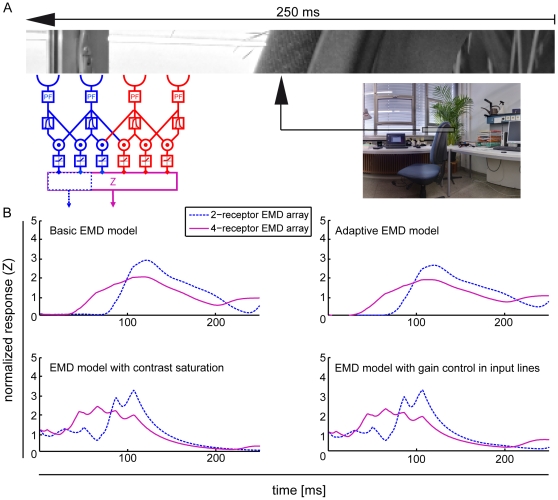
Pattern dependent response modulations of EMD models. (A) Image III (Fig. 2A) sampled by a one-dimensional EMD array, with either 2 (blue) or 4 (blue and red) receptors integrated. Image translates horizontally for 300 ms with a speed of 60°/s in preferred direction. (B) Normalized EMD responses Z for all models (Fig.1A–D) corresponding to the marked region in the input image. Blue response traces correspond to an EMD array integrating 2 receptors and pink response traces to an array integrating 4 receptors. pattern-dependent modulation amplitude and temporal response characteristics differ between models.

All LPTC models show pattern-dependent modulations coupled to the position of the input image (Fig. 3). The responses of single EMDs depend on prominent features of the moving panoramic image data used as visual input. The marked region in the input image and the corresponding normalized (see Methods and Materials) EMD array responses integrating one (blue) or three (blue + red) EMDs show examples of this dependency: due to the relatively high contrast step from background to the chair in the input image, the models respond with a strong increase in relative response amplitude. By increasing the number of spatially integrated input channels pattern-dependent modulation amplitude and smoothness change. Although all models show pattern-dependent modulations dependent on the contrast distribution and the number of integrated receptor channels, the modulation amplitude and the temporal response characteristics differ between models (data not shown). The pattern-dependent modulation amplitudes reach different maximum values for all models. Also the onset and decay of the modulations differ between models. These differences in maximum amplitude and temporal response characteristics indicate that the specific features of the different models influence the pattern-dependent modulations of the simulated LPTC response.

### One-dimensional receptive fields

**Figure 4:**
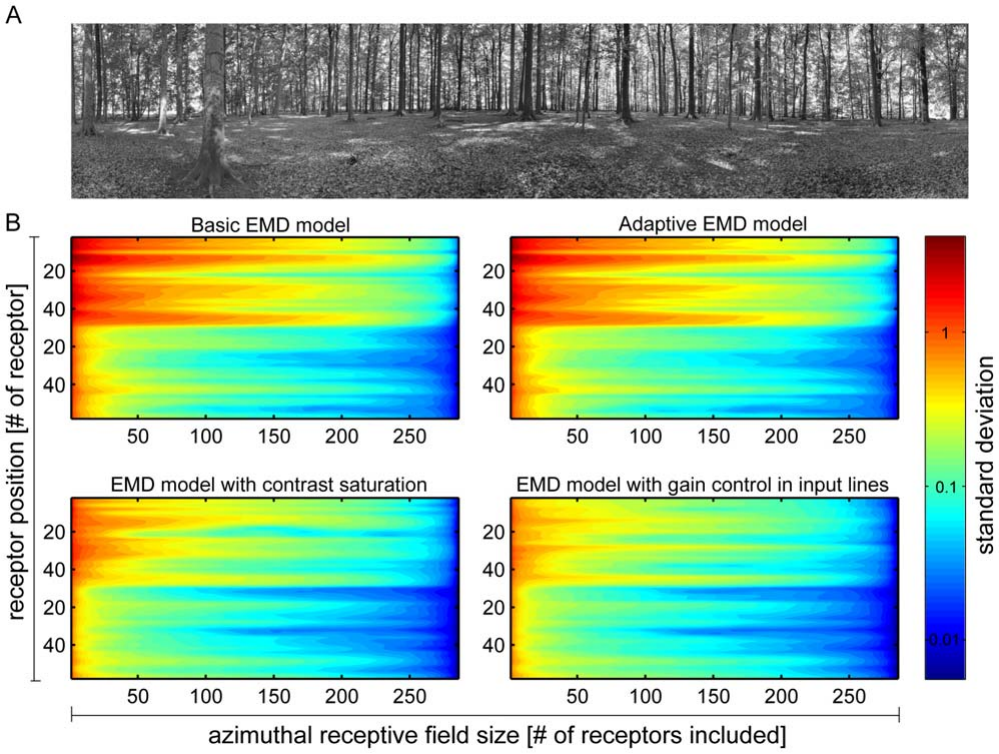
Pattern-dependent modulations of EMD models with one-dimensional receptive fields. (A) Panoramic high dynamic range input image II (Fig. 2) used exemplarily for stimulation. (B) Logarithmic color coded standard deviation describing the mean pattern-dependent modulation for one-dimensional receptive fields differing in vertical receptor position and azimuthal receptive field size (# of receptors included horizontally) for all models. In all models pattern-dependent modulation amplitude decreases with horizontal receptive field extent. With increasing receptive field extent pattern-dependent modulations are reduced to a higher extent in models with contrast saturation (C&D). Further, pattern-dependent modulation amplitude depends on the contrast distribution of the input image, as can be seen, when comparing pattern-dependent modulation amplitudes corresponding to the upper (trees) and lower part (ground) of the input image.

To compare the degree of pattern-dependence of model LPTCs with different receptive field sizes, the pattern-dependent modulation amplitude was quantified by computing their standard deviation over time. Figure 4 shows the standard deviations of simulated normalized responses of a one-dimensional EMD array at different elevations of a sample image. The horizontal extent of the one-dimensional EMD array was varied between 1 EMD and 288 EMDs (corresponding to an angular extent of 1.25°–360°). For each model version the standard deviation of the pattern-dependent modulations decays with increasing horizontal extent of the EMD array for all elevations. The maximum amplitude and the reduction of the pattern-dependent modulation amplitude depend on the elevation of the one-dimensional receptive fields. This can be seen, when comparing the standard deviations, for instance, in the elevation range ∼1–30 (corresponding to the sky in the input image) with the standard deviations in the elevation range ∼30–50 (corresponding to the ground): Maximum amplitudes differ according to the contrast ranges in the corresponding image regions. This complies with the data shown in figure 3, as differing local contrasts along different horizontal sections of the input image lead to differences in the response amplitudes.

Although mean pattern-dependent modulation amplitudes of all models decay with the number of receptors included in the array, the range of values and the reduction of the modulations with increasing array extent differ between models. This is most obvious when comparing the standard deviations for the different models. The functional modifications added to the different models reduce pattern-dependent modulations to different extents. The rate of decay changes most strongly when contrast saturation is included in the input lines of the movement detectors. This can be seen, when comparing the color-coded pattern-dependent modulations of the basic and the adaptive EMD model responses (Fig.1A,B) with the response modulations of the EMD model with contrast saturation and the EMD model with gain control in input lines (Fig.1C,D): with increasing receptive field size pattern-dependent modulations are reduced to a higher extent, when saturating non-linearities or gain control in the input line of the EMDs are included in the model as compared with the two other model versions.

### Two-dimensional receptive fields

**Figure 5 pone-0021488-g005:**
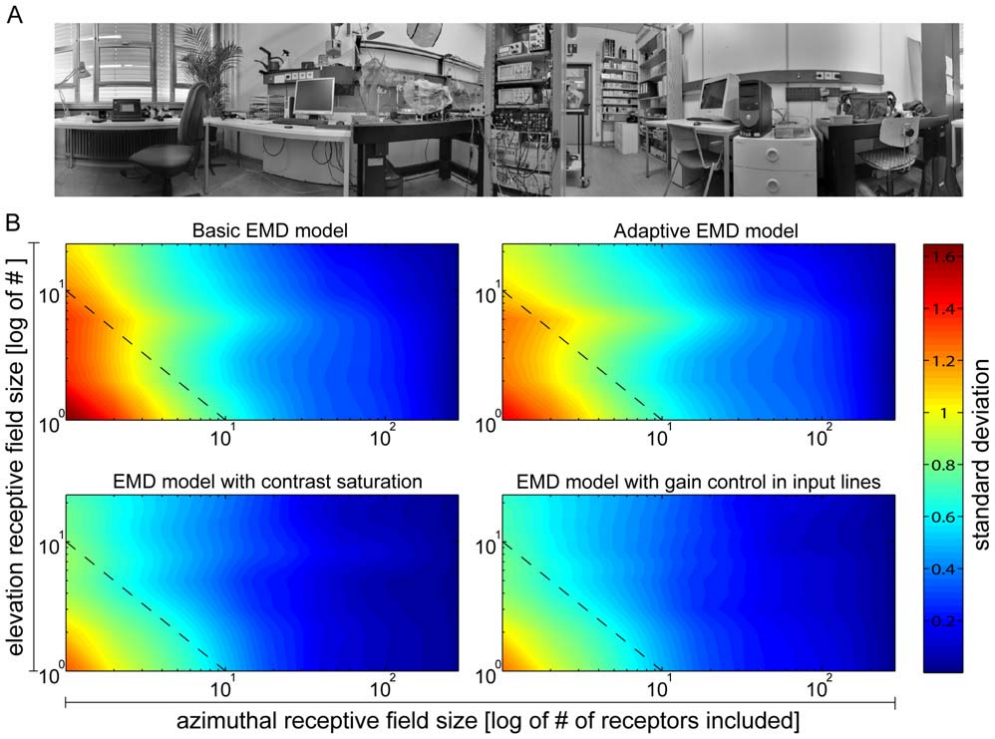
Pattern-dependent modulations of EMD models with two-dimensional receptive fields. (A) Panoramic high dynamic range input image used exemplarily for stimulation. (B) Color coded standard deviation describing the mean pattern-dependent modulation for two-dimensional receptive field arrays for all models. Receptive field size is defined via the number of receptors included in the integration of EMD signals in elevation and azimuth. Two-dimensional receptive fields are achieved by expansion of a one-dimensional receptive field located at the center of horizon in its vertical and horizontal size. The iso-line describes exemplarily receptive field sizes with 10 receptors included. The cross corresponds to a square receptive field (m = n) with 256 receptors included.

Figure 5 shows the mean amplitude of pattern-dependent modulations for two-dimensional EMD arrays exemplarily for one input data set. For convenience, we only simulated rectangular receptive fields. The size of the EMD array is defined by the overall number of receptors included in the integration, i.e. 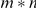 receptors with *m* the number of vertical and *n* the number of horizontal EMD signals. EMD arrays were centred on the vertical axis of the input image during sampling. The decay in amplitude with increasing size of the EMD array is visualized on a logarithmic scale for the vertical and horizontal extent of the receptive field (defined by log number of receptors integrated in the receptive field). For all models, the pattern-dependent modulation amplitude decreases with increasing two-dimensional array size. This can be observed for size changes in the horizontal, as well as in the vertical direction. This effect is most pronounced for the models with some kind of contrast normalization in the detector input lines, i.e. the EMD model with contrast saturation and the EMD model with gain control in input lines (Fig.5 bottom panels).

### Aspect ratio of receptive fields

In the basic and the adaptive EMD model the decay of pattern-dependent modulations depends on the aspect ratio of the integrated array, since an enlargement of the receptive field along the elevation and the azimuth of the visual field affects their standard deviation in different ways (panels in the middle of Fig.5). Moreover, in particular for the basic model and the adaptive model pattern-dependent modulations decrease more effectively, if the receptive field is elongated along the horizontal axis, i.e. along the direction of pattern motion, than when it has a more compact form (e.g. a square; compare the data points on the diagonal line corresponding to receptive fields of equal size). However, when saturating non-linearities are included in the LPTC models (Fig. 1C&D), this effect is no longer prominent, indicating that the aspect ratio of the receptive field plays only a relatively small role in influencing the pattern-dependent modulation amplitude in the model with contrast saturation and the model with gain control in input lines.

**Figure 6:**
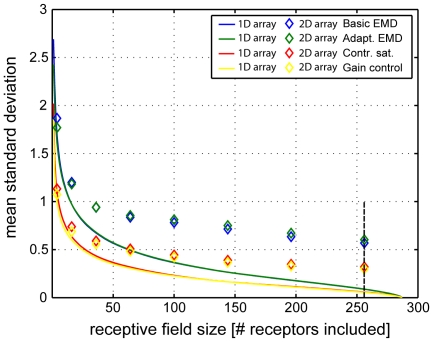
Mean pattern-dependent modulations for one- and two-dimensional EMD array responses. Mean pattern-dependent modulations over all input images for the different (color-coded) models: blue  =  *Basic EMD model*, green  =  *Adaptive EMD model*, red  =  *EMD model with contrast saturation* and yellow  =  *EMD model with gain control in the input lines*. Solid lines correspond to pattern-dependent modulations of one-dimensional EMD array responses, symbols correspond to responses of square EMD arrays. Mean pattern-dependent modulations decay stronger with increasing receptive field extent in one-dimensional EMD arrays, compared to square arrays. Black dashed line indicates receptive field size with 256 receptors integrated.

To investigate the influence of the aspect ratio of the EMD array on the pattern-dependent modulation amplitudes, we computed their standard deviations for each model and for all input images. Figure 6 shows the mean standard deviation of pattern-dependent modulations for one-dimensional EMD arrays (colored lines) and square two-dimensional EMD arrays (colored symbols) for all images. Pattern-dependent modulation amplitudes decay with increasing receptive field size for horizontal one- as well as two-dimensional EMD arrays. The models with saturating non-linearities and gain control in the input lines (Fig. 1C&D) show a steeper decay in mean pattern-dependent modulations with increasing array size. This can be observed for both one- and two-dimensional EMD arrays and complies with the data depicted in figure 4 & 5.

The decrease in pattern-dependent modulations in all models depends on the aspect ratio of the array. One-dimensional arrays show a relatively strong decay of amplitudes with increasing number of receptors integrated horizontally, i.e. along the direction of motion. The decay achieved by extending two-dimensional EMD arrays is less effective. This becomes obvious when comparing the decay of pattern-dependent modulations between receptive fields differing in the number of receptors included. Pattern-dependent modulations for one-dimensional arrays with 256 receptors (see Fig. 6) are reduced, on average, for all models by approx. 97%, when compared to an EMD array with only two receptors. For a same-sized square array (shown as + in Fig. 5) in the basic EMD model and the adaptive EMD model modulations are reduced on average by only approx. 71% and in the EMD model with contrast saturation and the EMD model with gain control in input lines on average by approx. 78%.

### Estimated HSE cell receptive field

**Figure 7:**
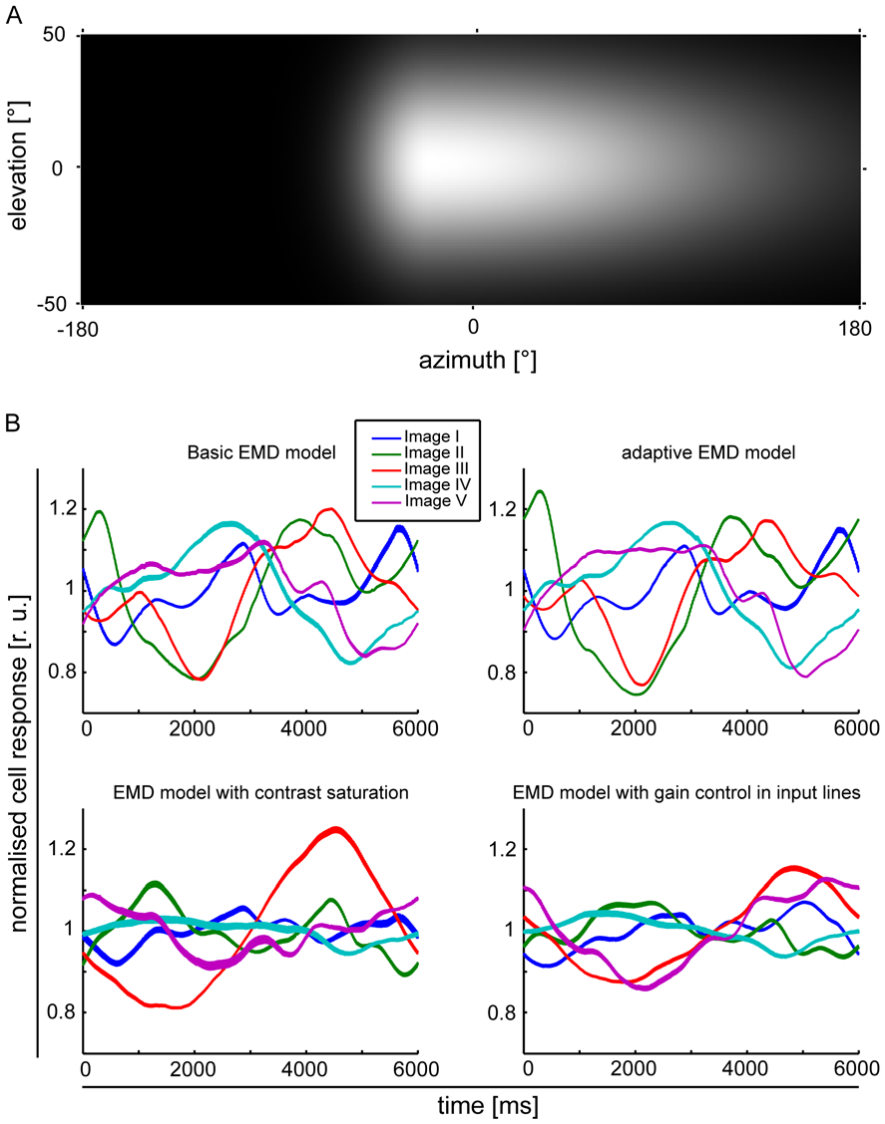
EMD array responses with an estimated HSE cell receptive field. (A) Weight field estimate of the spatial sensitivity distribution of a model HSE cell. The brighter the gray level the larger the local weight of the corresponding EMDs and, thus, the spatial sensitivity. The frontal equatorial viewing direction is at 0° azimuth and 0° elevation. (B) Normalized response traces of HSE models with the four types of EMD variants as indicated in the figure. Image motion was performed for 12s in preferred direction with an angular velocity of 60°/s. Responses to all image datasets are shown.

Figure 7 shows the normalized responses of the model EMD arrays, weighted with an estimated HSE cell receptive field. This HSE model has been successful in accounting for experimentally determined time-dependent HSE responses even to complex natural optic flow patterns as flies experience during free flight [19]. Here the HSE model was stimulated with constant velocity horizontal motion of the panorama images in the cell's preferred direction. Response traces of the HSE model with basic EMDs and with adaptive EMDs modulate in the amplitude range of approximately +/−0.2 relative response units. Standard deviation for the basic EMD model is 0.099 and 0.106 for the adaptive EMD model, respectively. The pattern-dependent modulations and the temporal response characteristics between both models differ only slightly.

Pattern-dependent modulation of the HSE model with EMDs containing contrast saturation or gain control in the input lines (Fig. 1 C&D) are reduced compared to the models lacking contrast saturation; except for the response trace for image III of the EMD array with contrast saturation, the modulation bandwidth is approximately +/−0.1 relative response units. The standard deviation for the response trace of the EMD array with contrast saturation is 0.062 and 0.058 for the EMD model with gain control in the input lines. Whereas pattern-dependent modulations and temporal characteristics differ slightly between these two models, more pronounced differences can be seen in the responses of EMD arrays lacking contrast saturation.

## Discussion

In insects the outputs of arrays of EMD-like motion-detectors are assumed to be spatially integrated by LPTCs. Different types of LPTCs have been associated with a variety of different tasks based on optic flow processing [2]. When HS cells, one type of LPTCs, are stimulated visually with moving natural images, the responses were found to depend on motion velocity. Nonetheless, the responses also show pronounced pattern-dependent response modulations. Such modulations are mimicked by our LPTC models. The pattern-dependent modulations reflect the structure of the visual scene. Although the responses of LPTCs increase with contrast in the low contrast range [15], they are relatively invariant to the significantly differing global contrast values characteristic of natural images [21]. Mechanisms that may contribute to this relative contrast invariance have been proposed [15], [8], and some of these features have been implemented in the LPTC model versions used in our study.

We show that the number of EMD outputs integrated by LPTC models influences pattern-dependent response modulations to a great extent (Fig. 6), as spatial changes in contrast and spatial wavelength within the visual field are smoothed out by the integration of many phase-shifted EMD outputs. The pattern-dependent modulations decay with increasing number of integrated EMD outputs. Small changes in receptive field size lead to strong effects in the pattern-dependent modulations of LPTC models for relatively small receptive fields. For larger receptive field sizes a much larger increase is required to achieve a similar decrement in the pattern-dependent modulations.

Furthermore, the aspect ratio of the receptive field influences pattern-dependent modulations in the responses of LPTC models, especially if the EMDs do not contain elements in their input lines that normalize contrast in some way (Fig. 6). A similar dependency has also been described by Rajesh and colleagues [7]. In the models including a kind of contrast saturation in the peripheral processing stages, the relation between aspect ratio and pattern-dependent modulations is no longer prominent. Contrast saturation reduces the modulations and their decay with increasing receptive field size, as the contrast range is computationally compressed. Consequently, responses become more invariant with respect to variations in pattern contrast [15]. However, even then pattern-dependent response modulations may be very pronounced. The influence of receptive field shape on pattern-dependent modulations in the models without contrast saturation is likely to be the consequence of differences in the variations in pattern contrast along the horizontal and the vertical axis of the input image, respectively. When considering the images of natural environments used for simulation, contrast distributions and image statistics may differ considerably in the upper and the lower part of the images (for instance, due to differences in the structure of sky and ground). In the simulations of the consequences of the aspect ratio of the LPTC models' receptive fields, their location was centred at an elevation close to the horizon of the input images. Hence, a differential extension of the receptive fields along the horizontal and vertical axis of the visual field, respectively, may lead to different pattern-dependent modulations due to different variations in pattern contrast along the two axes. When contrast saturation is included, this effect is reduced, as the model response is more invariant to the differing local pattern contrasts in the upper and lower part of the input images, respectively.

In contrast to saturation-like non-linearities in the input lines of EMDs (Fig.1C&D), the other computational elements additionally included in the EMD models affect pattern-dependent modulations only to a relatively small degree. When the basic model (Fig 1A) is elaborated by adaptive elements as proposed by Borst and colleagues [14] (Fig. 1B), mean pattern-dependent modulations are reduced only relatively little (Figs.4-6; see also [8]). However, this EMD variant was previously shown to affect the transient modulations of the step response while having only a small influence on the steady-state velocity tuning [14]. Consequently the influence of adaptive EMDs on pattern-dependent modulations is relatively small for the constant-velocity stimulus used in the present study.

The sensitivity distribution within the receptive field of biological LPTCs differs from that of the LPTC models we studied systematically with respect to their consequences for pattern-dependent modulation amplitudes. In LPTCs the spatial sensitivity distribution of the receptive field is not constant, but may vary considerably in a graded way. To analyze the consequences of such a spatial sensitivity distribution we simulated the different LPTC model variants after the EMDs subserving the receptive field were weighted according to the sensitivity values measured experimentally for HSE cells. Although having a relatively large receptive field [17], [18], the HSE cell model shows pronounced pattern-dependent modulations when stimulated with natural images (Fig. 7). The amplitudes of the modulations of the HSE model are affected by the EMD variants included into the model in a similar way as was observed for the model LPTCs with constant spatial sensitivity distribution. Depending on the EMD model, the range of pattern-dependent modulations of the model HSE cell varies between 20% and about 40% of the mean response. These results suggest that a receptive field with a sensitivity peak and a sensitivity tapering out towards the edges of the receptive field leads to larger modulation amplitudes than receptive fields with the same size, but constant sensitivity distribution.

Irrespective of the details of how pattern-dependent modulations depend on particular model features, one general conclusion can be drawn from our model simulations. Receptive field size is subject to a trade-off when considering velocity coding and localization of pattern motion in the visual field: Large receptive fields, on the one hand, improve the quality of velocity signals, however, at the expense of their locatability. On the other hand, if motion signals need to be localized by a neuron, its receptive field should be sufficiently small; then, however, velocity coding is only poor, but the signal provides – potentially useful – local pattern information. This trade-off suggested by our modelling results, thus, hints at an uncertainty in motion representation by correlation type movement detectors: you cannot obtain both a good and a localized velocity signal from the output of a single cell. Hence, the size and geometry of receptive fields should be adjusted according to the particular task of the motion sensitive neuron: they should be large, if velocity signals without pattern-dependent modulations are required, but should be relatively small, if motion dependent pattern information is required that can be localized in the visual field.

## Materials and Methods

### Input data sets

Five different panoramic high dynamic range images reflecting possible habitats of flies and differing in spatial clutter, contrast and luminance were used as input data sets (see Fig. 2). In each scene a series of 30 color photographs was taken using a digital single-lens reflex camera (Canon EOS 450D) equipped with a rectilinear object lens (Canon Zoom Lens EF-S 18–55 mm 1∶3.5–5.6 IS). The camera was rotated longitudinally about the nodal point of the lens at 36° intervals via a panoramic tripod attachment. At each interval three images were taken at different exposure levels (−2.0, 0 and +2.0EV bracketing) in order to capture details exceeding the dynamic range of the cameras CMOS chip. Focal distance and aperture were fixed within scenes. The images were stored in an 8-Bit high-quality JPG format. The corresponding images were ‘stitched’ to high dynamic range panorama images using the open-source software toolbox *Hugin* (http://hugin.sourceforge.net/; author: Pablo d'Angelo; licensed under GPL2). Due to the temporal latency between captures, movements of details in the scene might have resulted in spatial low-pass filter effects ('ghosting' or 'blurring') in image scenes with different exposure levels or overlapping image regions. Furthermore, spatial corrections for lens distortions and software alignment of the panels to produce panoramas may have reduced the overall detail of the panorama scenes. The vertical extent of each scene after stitching ranged between approx. −35° to +35°, with the horizon at center. After down-scaling the final sizes of the images varied in the range of approximately 8000 by 1600 pixels.

### Models

The compound eye of flies consists of a two-dimensional array of hexagonally aligned ommatidia comprising the retina. Each ommatidium contains a lens and a set of photoreceptor cells. After photo-transduction in the retina, luminance signals in the visual motion pathway are processed by three successive optical ganglia: the lamina, medulla and lobula complex. Motion-sensitive LPTCs reside in the lobula plate, a substructure of the lobula complex [22], [23]. According to the functional operations performed and the physiological counterparts in the fly visual system each of the models analyzed in this study can be divided into three successive stages comprising the visual motion pathway up to the LPTCs: a) input stage, (b) correlator stage, (c) integration stage. The input stage pre-processes luminance changes in the input signals by elements corresponding to the photoreceptors and their post synaptic elements in the retina and lamina. The correlator stage is built by local elementary motion detectors (EMDs) that are fed by the pre-processed luminance signals and are believed to reside in the medulla of the fly visual system. The basic EMD consists of two mirror symmetrical units, the so-called half-detectors. In each unit the time-delayed signal from one unit is multiplied with the un-delayed signal from the complementary unit (Fig. 1). At the integration stage, the motion detector outputs are spatially pooled by elements corresponding to the dendrites of LPTCs in the lobula plate. Therefore LPTCs will have their maximum response for signals that have a distinct temporal delay in a distinct direction. These three processing stages are common to all model variants tested here (Fig. 1). All computational models were programmed in MATLAB (The Mathworks, Natick, MA, USA). Model parameters were adjusted according to [8], if not stated differently. A basic EMD model formed the reference for the more elaborated models.

### Common computational elements of all model versions

The basic model (Fig. 1A) includes temporal filters in the EMD input lines to mimic the dynamic properties of cells in the retina and lamina, correlational EMDs, and nonlinear integration by the model LPTC in the form of the “gain control” model [13].

#### Input stage

Fly compound eye optics have characteristics of a spatial low-pass filter and blur the retinal image. The extent of filtering is matched to the inter-ommatidial angles to avoid spatial aliasing [24]. To mimic the spatial filtering of the eye, input panorama images were sampled by a two-dimensional Gaussian-shaped spatial low-pass filter *F* according to [8]:
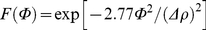
(1)


The inter-ommatidial angle *Φ* between equally spaced direct photoreceptor neighbours was set to 1.25° and the acceptance angle of the ommatidium *Δρ* to 1.64° in order to approximate the characteristics of the blowfly eyes [25]. Although blowflies in general possess color vision, evidence suggests that the pathways involved in motion detection are monochromatic [26]. Therefore only the green color channel of the high dynamic range panorama images was used for visual stimulation. After pre-processing each input image was scaled to luminance values on a rectangular grid of photoreceptors [19]. The photoreceptor grid covered a visual field of 360° horizontally. Visual motion was simulated by stepwise displacement of the input image in horizontal direction with a sample rate of 1 kHz. The luminance changes due to displacement of the input image at the subsampled image coordinates were computed via bilinear interpolation between luminance values of neighbouring samples.

Phototransduction in the receptors of the compound eye has been shown to be nonlinear: the receptor membrane potential depends on luminance in a logarithmic manner based on a working point adapted to luminance history [27] to account for differing dynamic contrast ranges in different environments (as reflected by the different high dynamic range panorama images). These characteristics were achieved by a Naka-Rushton transformation [28], [8]: 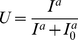
(2)where *I* is the input intensity and *I_0_* the mid-response intensity level. The mid-response intensity level *I_0_* was set according to an estimate of the geometric mean of the luminance over the corresponding input images. The parameter *a* defining the slope of the transfer function was set to 0.7 [8].

In the visual pathway of the fly the output signals of each receptor is processed by *lamina monopolar cells* (LMCs) located in the first optic ganglion. To mimic the temporal band-pass-like response characteristics of the LMCs [29], a temporal band-pass filter function was included in the input stage. The transfer function was implemented via serially aligned recursive first-order low-pass and high-pass filters with the iterative approximation
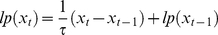
(3)for the low-pass filter and
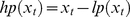
(4)for the high-pass filter, respectively. The high-pass filter time-constant τ_H_ was set to 400 ms and the low-pass filter time-constant τ_L_ to 8 ms [8].

#### Correlator stage

Elementary motion detection in the fly visual system is assumed to take place primarily in the medulla [23]. Motion detection can be modelled based on the multiplication of the delayed signal of one receptive unit with the un-delayed signal originating from a neighboring unit [3]–[5], [30]. In our model EMDs only interactions between nearest neighbors in the ommatidial array are taken into account. The delay operator τ_lp_ in each half-detector was modelled by a temporal first-order low-pass filter with a time-constant τ_lp_ of 40 ms [31].

#### Integration stage

The integration of EMD output signals is assumed to be performed by LPTCs in the lobula plate of the fly. The dendrites of LPTCs cover wide areas of the lobula plate [4]. Dendritic integration of LPTCs is modelled by integrating the outputs of differently shaped arrays of EMDs with horizontal preferred direction. The integration is not linear, but is accomplished according to physiological findings on the basis of a *gain control* mechanism [13]. This mechanism normalizes the spatial sum of half-wave rectified EMD inputs for different stimulus extents as an effect of synaptically varied and constant leak membrane conductances. The interaction of excitatory and inhibitory signals results in the strongly directional LPTC response integrating weakly directional EMD outputs. The response of the integrated EMD arrays of all model variants are given by
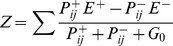
(5)where *Z* is the cell response and *E^+^* and *E^−^* the depolarizing and hyperpolarizing reversal potentials. It is supposed that depolarizing and hyperpolarizing classes of inputs correspond to the outputs *P^+^* and *P^−^* of complementary pairs of half-detectors. The index *i (i = 1...n)* designate the principal directions with which EMDs are aligned and *j (j = 1...m)* indicate the position of the EMDs within the receptive field area of a tangential cell. *G_0_* describes the fixed membrane leak conductance. For reasons of simplicity *E^+^* and *E^−^* and *G_0_* were set to 1 resulting in 
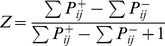
(6)in order to simulate the membrane potential Z in the model.

#### Estimation of an HSE cell receptive field

The receptive fields of HSE cells cover regions of up to approx. 120° in the azimuth and 40° in the elevation of the fly's eye [17], [18]. However, the sensitivity distribution within the receptive field of the HSE cell is not constant. Rather, the sensitivity declines dramatically towards the edges of the receptive field. Different local sensitivity distributions correspond to different weightings of EMD outputs during summation by the LPTC. The receptive field of a HS cell was estimated by weighting the EMD outputs *P^+^_i,j_* and *P^−^_i,j_* with a factor *w* using a two-dimensional Gaussian filter function of the form.
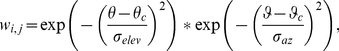
(7)where ϕ_c_ ( = −15°) represents the azimuth and θ_c_ ( = 2°) the elevation of the center of the receptive field. The vertical extent of the receptive field is determined by σ_elev_ which was set to 35° and the horizontal extent given by σ_az_ was set to 120° for (*θ*–*θ_c_*)>0 (i.e. in the lateral part of the receptive field) and 25° (*θ*–*θ_c_*)<0 (i.e. in the frontal region of the receptive field). Zero degree corresponds to the frontal equatorial direction. Receptive field data was estimated according to [19].

### The different model variants

#### Basic EMD model (Fig.1A)

The basic EMD model consists of the computational elements common to all tested model variants as described above without further elaborations.

#### Adaptive EMD model (Fig.1B)

When LPTCs are stimulated by an abrupt onset of constant motion, transient response oscillations can be observed [32], [15], [33]. The values experimentally determined for the duration of these oscillations in different adaptive states of LPTCs differ from those in EMD models with only one delay filter. To resolve this problem, the basic EMD model was extended by inserting a high-pass filter in the cross-arms of the correlator stage (Fig. 1B, [14]). By dynamically adjusting the high-pass filter time-constant according to the low-pass output of the contralateral arm of the EMD, the authors additionally implemented a mechanism for adaptation. Via this extension the influence of the high- and the low-pass filter on various response properties of the adaptive EMD model decouple in such a way that the shortening of the high-pass filter time-constant strongly affects the transient response oscillations of the step response while having small influence on the steady-state velocity tuning [14]. The adaptive EMD model is implemented via the extension of the correlator stage of the basic EMD model with a first order high-pass filter (see transfer function 3) in the cross-arms of the EMD. The time-constant of the high-pass filter τ_h_ is adjusted over time according to
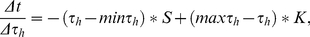
(8)where *S = lp(|L*'*|)* represents the rate of change of the corresponding low-pass signal (τ_S_ = 500 ms). L' is the first derivative of the low-pass filtered luminance signal. *min_τh_* and *max*_τh_ define the range in which τ_h_ is adaptive and *K* a constant relaxation factor. K and S define the speed of adaptation proportional to the position of τ_h_ in the range defined by min_τh_ and max_τh_. The modelling results were obtained for max_τh_  = 500 ms and min_τh_  = 0 ms. The relaxation constant K was set to 0.1 kHz [14].

#### EMD model with contrast saturation (Fig.1C)

Insect EMDs show a saturating contrast response curve, which can be accounted for most parsimoniously by introducing saturating non-linearities [15]. Consequently the basic model was extended by including saturating non-linearities in the input lines of the EMDs (Fig. 1C).

Saturation of early vision signals (*s*) was modelled with multiplication by a scaling factor *a* followed by application of a hyperbolic tangent function of the form:
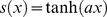
(9)


To quantify the degree of saturation, *a* was defined according to 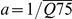, where 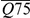 was the mean of the 3rd quartiles (75% of data included) of all input signals after pre-processing by previous model compartments.

#### EMD model with gain control in the input lines (Fig.1D)

When exposing the visual system of an intact animal to strong motion stimuli, subsequently probed with small impulses or steps in velocity, the response of a tangential cell is reduced relative to that of the same cell before motion stimulation [34], [35], [32]. This phenomenon – termed motion adaptation – is implemented via a mechanism for controlling the gain in the input lines of the EMDs in order to reduce contrast dependence (Fig. 1D, [8]). The mean absolute deviation of each input signal is estimated via full-wave rectification, followed by a linear, first-order low-pass filter (transfer function see 2) with time-constant τ_A_. The input from each channel is divided by the mean absolute deviation, and τ_A_ is a measure of the time scale of adaptation and was set to 200 ms.

### Data acquisition

The response *Z* of the different model variants to horizontal constant velocity motion of the panorama images was computed for different sizes of EMD arrays. Image motion was performed for *t* = 12 s in preferred direction with an angular velocity *v* = 60°/s, resulting in two 360° horizontal image rotations. *v* was set to the velocity response optimum of the EMD model according to [8]. This parametrization additionally comprised a good trade-off between stimulation duration and computing time. The sizes of EMD arrays (*m*-by-*n*) were adjusted by the number of correlator outputs *P^+^* and *P^−^* included horizontally and vertically in the computation. To exclude transient effects at the onset of motion and thus to take only non-transient responses into account, the initial 6 s of stimulation time (i.e. the first 360° rotation of the input dataset) were discarded.

#### Data analysis Normalization

To compare the simulated responses of the different models variants, their output was normalized in the form of
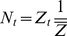
(10) where *N_t_* represents the normalized cell output at time *t,* 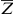 is the mean cell response over time.

#### Standard deviation

The influence of different receptive field sizes on pattern-dependent modulations in the normalized response was quantified by computing the standard deviation *s* from the mean response according to
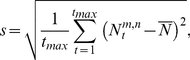
(11)where *t* represents time and *N^m,n^* is defined as the normalized cell response with a receptive field size of *m*-by-*n*. 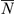 is the cell response with a receptive field comprising all receptive channels, corresponding to the integration of the full input image set at each time step.

#### Root mean square contrast

To estimate the global contrast of the input panoramas, the root mean square (RMS) contrast 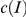 was calculated by dividing the standard deviation 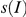 of luminance values by the global luminance mean 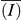 of each image 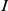:
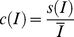
(12)

